# Functional Genetic Diversity and Plant Growth Promoting Potential of Polyphosphate Accumulating Bacteria in Soil

**DOI:** 10.1128/spectrum.00345-21

**Published:** 2022-02-23

**Authors:** Sonal Srivastava, Vandana Anand, Jasvinder Kaur, Manish Ranjan, Vidisha Bist, Mehar Hasan Asif, Suchi Srivastava

**Affiliations:** a Division of Microbial Technology, CSIR-National Botanical Research Institute, Lucknow, India; b Academy of Scientific and Innovative Research, AcSIR, Ghaziabad, India; c Department of Botany, Kumaun University, Nainital, India; d Computational Biology Laboratory, Genetics and Biotechnology Division, CSIR-National Botanical Research Institute, Lucknow, India; University of Massachusetts Amherst

**Keywords:** *Arabidopsis thaliana*, functional diversity, genetic diversity, phosphate accumulating bacteria, plant growth promoting rhizobacteria, rhizosphere soil, salinity stress

## Abstract

Polyphosphate (polyP) accumulation is an important trait of microorganisms. Implication of polyP accumulating bacteria (PAB) in enhanced biological phosphate removal, heavy metal sequestration, and dissolution of dental enamel is well studied. Phosphorous (P) accumulated within microbial biomass also regulates labile P in soil; however, abundance and diversity of the PAB in soil is still unexplored. Present study investigated the genetic and functional diversity of PAB in rhizosphere soil. Here, we report the abundance of Pseudomonas spp. as high PAB in soil, suggesting their contribution to global P cycling. Additional subset analysis of functional genes i.e., polyphosphate kinase (*ppk*) and exopolyphosphatase (*ppx*) in all PAB, indicates their significance in bacterial growth and metabolism. Distribution of functional genes in phylogenetic tree represent a more biologically realistic discrimination for the two genes. Distribution of *ppx* gene disclosed its phylogenetic conservation at species level, however, clustering of *ppk* gene of similar species in different clades illustrated its environmental condition mediated modifications. Selected PAB showed tolerance to abiotic stress and strong correlation with plant growth promotary (PGP) traits *viz.* phosphate solubilization, auxin and siderophore production. Interaction of PAB with *A. thaliana* enhanced the growth and phosphate status of the plant under salinity stress, suggestive of their importance in P cycling and stress alleviation.

**IMPORTANCE** Study discovered the abundance of Pseudomonas genera as a high phosphate accumulator in soil. The presence of functional genes (polyphosphate kinase [*ppk*] and exopolyphosphatase [*ppx*]) in all PAB depicts their importance in polyphosphate metabolism in bacteria. Genetic and functional diversity reveals conservation of the *ppx* gene at species level. Furthermore, we found a positive correlation between PAB and plant growth promotary traits, stress tolerance, and salinity stress alleviation in *A. thaliana*.

## INTRODUCTION

Both animal and plant system cannot function without interacting with their microbial partners. These plethora of microbial partners colonize the host plant and regulate different host functions (1). They play numerous roles in maintaining health of the host plant by involving differential behavioral strategies, as per environmental requisite. Cognizance reveals very close interaction of the microbes and plants at the rhizosphere. This plant microbial niche influences plant biogeography and ecosystem function by enhancing nutrient availability through their cycling.

Phosphorus (P) being a key macronutrient play an important role for the growth and sustenance of the plant. Availability of inorganic phosphate in soil is suboptimal due to its high reactive nature. P solubility decreases due to fixation of P in the form of aluminum, iron, or calcium phosphates. Root associated microbiota affects P cycling by exhibiting unique enzymatic and metabolic activities. The role of soil microbes in P mobilization through diverse mechanism of solubilization and mineralization is well known (2–4). On the contrary, soil P is also immobilized by the rhizosphere microbes as temporarily unavailable P pool, which accounts for 2% to 10% of total soil P (5, 6). Immobilization of labile P within microbial biomass is an important mechanism for maintaining P supply in soil solution (7, 8). Therefore, P accumulating microbes are one of the key players in P cycling for its availability to plants over a time. Arbuscular mycorrhizal fungi (AMF) efficiently absorb P from soil and accumulate them as polyP. They effectively transfer P from soil to plant through appressoria and increase its uptake under phosphate deficient condition (9, 10). Tandon et al. (11) showed accumulated polyphosphate (polyP) granules in P solubilizing *Trichoderma koningiopsis* through microscopy.

Accumulation of P in microbes occurs in the form of a linear polymer of orthophosphates, linked through high energy phospho-anhydride bond and serve as an energy source under P starved condition (12, 13). P accumulation in microbes is a two-step process: (i) synthesis by polyphosphate kinase (ppk) and (ii) degradation; i.e., release of Pi from the terminal P of polyp chain, by the action of exopolyphosphatase (ppx) enzyme under starved conditions. Polyphosphate kinase (*ppk*) is well known to be associated with the biofilm formation, virulence, motility, and stress tolerance of the microbes (14, 15). Application of polyP accumulating bacteria (PAB) in enhanced biological phosphate removal (EBPR) and heavy metal sequestration processes are known (16–18). Their genetic diversity associated with the function of phosphate accumulation under different conditions has been well documented (19–21).

Microbial diversity is critical to the functioning of different ecological processes. A strong positive correlation between the structure and function of soil microorganisms has been reported with some redundancy (22). Mutual interaction between plants and microbes is known to incur trade of resources in nature (23) in different biological, physiological, or ecological function. However, abundance of PAB in soil, their genetic and functional diversity, structural and functional correlation, and mutual interaction of these microbes with plant underpinning their triumph in soil niche is largely unknown.

Agriculture is challenged by many stresses, thereby escalating the problem of food security. Genetic engineering and other conventional plant breeding approaches have drawn flaws in their adoption from lab to field. Selection of a good microbial composition and microbiome engineering has been advocated as an advantageous approach for better plant growth than the single gene transfer. Numerous P solubilizing and mineralizing microbes are reported for alleviation of different abiotic stresses in plants (24–26). However, among the significant challenges along the road, studies with new microbial trait are indeed needed, as a viable option for crop productivity acceleration (27). Under such scenarios, sustainable agricultural practices that avail bacterial usage with polyphosphate accumulation properties open the avenues under increasing environmentally stressed conditions. High phosphate accumulating mutants of Arabidopsis thaliana has been shown to have increased tolerance to salinity stress (28). Additionally, salinity stress is also known to create P deprived condition in soil (29). Therefore, exploring their availability and diversity in different rhizosphere soil will develop a better understanding in terms of P availability and their correlation with plant growth promotion and stress alleviation for sustainable agriculture.

In this view, the present study was planned for the identification of bacteria involved in polyP accumulation to establish polyphasic taxonomy based on genotypic and biochemical data. Furthermore, single and combined markers-based analysis of the genetic and functional traits was performed to assess the discrimination efficiency of the genes for phylogenetic comparisons. Lastly, implication of PAB for salinity stress amelioration in model plant Arabidopsis thaliana has also been assessed to establish their correlation to stress amelioration and plant growth promotion.

## RESULTS

### Soil characteristics.

Soil samples collected from different regions showed a pH range between 7.29 and 7.83, depicting neutral to slight alkaline nature of the soil. However, differences in electrical conductivity (EC) among different soil were observed (Table 1). Shillong was found to have the lowest EC, i.e., 142.83 μS cm^−1^, while, maximum was recorded in soil of Raebareli (675.50 μS cm^−1^). Results showed variation in EC among different sites of Bulandshahr (112.95 to 564.50 μS cm^−1^), whereas, no such difference was observed in Deokhera and Punjab. Different levels of nitrogen (0.196 to 0.98 mg kg^−1^), phosphorus (0.322 to 2.21 mg kg^−1^), potassium (16.32 to 262.28 mg kg^−1^), and sulfur (6.75 to 117.81 mg kg^−1^) were recorded in soils of different sites. Regarding microbial biomass carbon (MBC), the highest value was found in Bulandshahr, site 3 (679.14 μg g^−1^), whereas, the lowest was noted in Shillong (145.53 μg g^−1^).

**TABLE 1:** Soil sampling sites with their chemical properties

Sampling sites	Sites	Avail. Nitrogen	Avail. Phosphorus	Avail. Potassium	Avail. Sulphur	MBC	pH	EC
		(mg kg^−1^)	(mg kg^−1^)	(mg kg^−1^)	(mg kg^−1^)	(μg g^−1^)		(μS cm^−1^)
Bulandshahr	Site 1	0.64 ± 0.02	0.35 ± 0.00	26.68 ± 2.6	11.73 ± 0.20	485.1 ± 97.02	7.65 ± 0.03	112.95 ± 0.55
	Site 2	0.42 ± 0.02	1.30 ± 0.01	136.04 ± 0.84	6.64 ± 0.16	679.14 ± 97.02	7.31 ± 0.01	564.5 ± 4.5
	Site 3	0.47 ± 0.02	1.24 ± 0.01	55.76 ± 0.56	52.85 ± 0.48	727.65 ± 48.51	7.75 ± 0.00	200 ± 0.1
	Site 4	0.64 ± 0.02	0.87 ± 0.00	90.8 ± 1.12	8.80 ± 0.20	312.08 ± 57.24	7.91 ± 0.00	186 ± 0.2
	Site 5	0.47 ± 0.02	0.85 ± 0.00	96 ± 0.08	10.83 ± 0.28	436.59 ± 145.53	7.24 ± 0.00	223.05 ± 0.15
	Site 6	0.36 ± 0.02	0.95 ± 0.04	26.8 ± 0.56	4.73 ± 0.44	291.06 ± 64.11	7.50 ± 0.00	205.3 ± 0.1
	Site 7	0.42 ± 0.02	0.61 ± 0.01	223.04 ± 6.24	20.76 ± 0.04	485.1 ± 194.04	7.49 ± 0.00	157.85 ± 0.05
	Site 8	0.58 ± 0.02	0.39 ± 0.00	16.32 ± 0.48	19.86 ± 0.20	727.65 ± 242.55	7.60 ± 0.00	267.85 ± 0.25
Raebareli	Site 1	0.58 ± 0.02	2.21 ± 0.00	262.28 ± 2.4	6.56 ± 0.08	367.08 ± 48.51	7.34 ± 0.01	675.5 ± 0.5
Deokhera, Rajasthan	Site 1	0.36 ± 0.02	0.59 ± 0.00	25.88 ± 1.4	11.64 ± 0.04	533.61 ± 242.55	7.28 ± 0.05	160.65 ± 0.45
	Site 2	0.98 ± 0.02	2.08 ± 0.01	137 ± 0.36	23.81 ± 18.63	291.06 ± 97.02	7.40 ± 0.00	155.65 ± 0.45
Punjab	Site 1	0.30 ± 0.02	0.41 ± 0.00	111.92 ± 0.6	9.41 ± 0.40	464.85 ± 124.06	7.22 ± 0.00	162.4 ± 0.1
	Site 2	0.36 ± 0.02	0.57 ± 0.01	66.24 ± 2.7	45.20 ± 0.89	630.63 ± 134.36	7.42 ± 0.00	200 ± 0.1
Gujarat	Site 1	0.19 ± 0.02	0.32 ± 0.00	26.16 ± 1.68	8.88 ± 0.20	339.57 ± 242.55	7.34 ± 0.01	185.85 ± 0.35
Shillong	Site 1	0.30 ± 0.02	0.38 ± 0.00	23.68 ± 0.56	117.81 ± 0.28	145.53 ± 48.51	7.55 ± 0.00	121.5 ± 0.1

### Screening of polyphosphate accumulating bacterial strains.

Based on qualitative screening, out of 446 bacterial strains, 46 bacterial strains were selected as phosphate accumulating bacteria (PAB). (Fig. S1; https://github.com/ssrivastava-nbri/Supplementary-file/blob/main/Supplementary%20file.pdf). Furthermore, accumulated P in selected bacterial strains was determined quantitatively. Based on accumulated P, these PAB were categorized as high (>150 μg/g biomass), moderate (100 to 150 μg/g biomass), and low (<100 μg/g biomass) P-accumulator. Among different sampling sites, high PAB majorly belonged to Deokhera followed by the Bulandshahr region of Uttar Pradesh, India (Fig. S2; https://github.com/ssrivastava-nbri/Supplementary-file/blob/main/Supplementary%20file.pdf).

### Distribution of plant growth promoting traits in phosphate accumulating bacteria.

Selected bacterial strains were characterized for different plant growth promoting traits *viz.* phosphate solubilization, biofilm formation, and production of auxin, siderophore, and phosphatase enzyme (Table S4; https://github.com/ssrivastava-nbri/Supplementary-file/blob/main/Supplementary%20file.pdf). Auxin production in bacterial strains ranged between 19.00 and 59.92 μg/mL, and highest auxin production was observed in bacterial strains (SSNBRI19) isolated from Gujarat. Similar levels of auxin production were observed in bacterial strains from Deokhera; however, phosphate solubilization potential of all bacterial strains varied irrespective of their isolation sites. Strains belonging to Bulandshahr, Deokhera, and Gujarat were also siderophore producers, while, isolates belonging to Punjab were deficient in siderophore activity. Among all the isolation sites, strains from Bulandshahr showed maximum acidic phosphatase enzyme production. However, alkaline phosphatase production was almost similar in all selected bacterial strains belonging to different isolation sites. Biofilm production in bacterial isolates ranged between 0.31 and 3.23.

**FIG 1:**
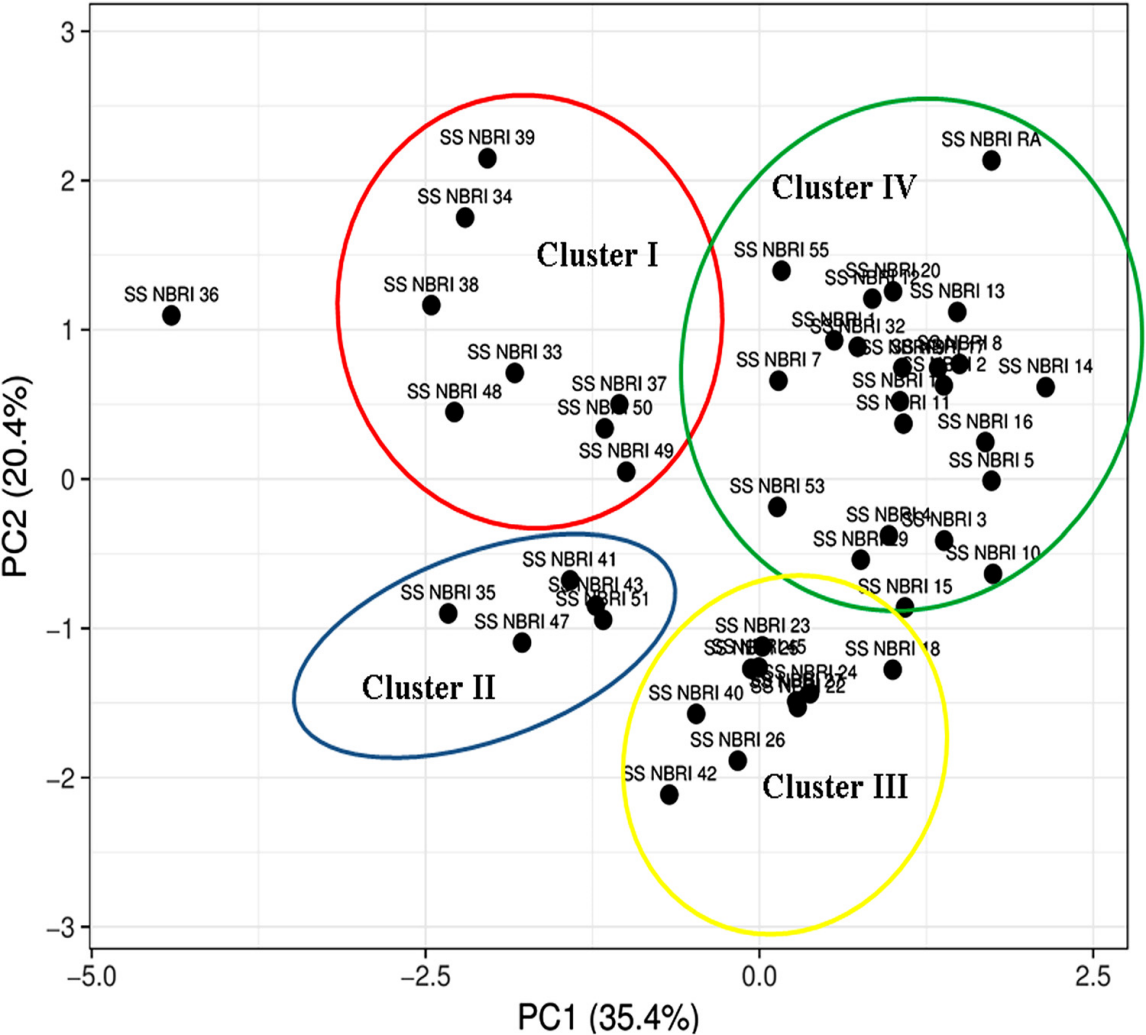
Correlation between phosphate accumulation with other plant growth promoting traits through principal-component analysis.

Relatedness among different PGP traits with P accumulation property was determined through principal-component analysis (PCA) (Fig. 1). The percent variability was higher due to PC1 factor (35.4%) compared with PC2 factor (20.4%). Based on the magnitude of plant growth promoting attributes, bacterial strains were clustered in four different clusters. Cluster I included bacterial strains having moderate auxin producing (20.23 to 35.39 μg/mL) and biofilm forming ability (3.43 to 5.95 O.D.) with high alkaline (10.69 to 46.55 μM pNP produced) and acidic (38.67 to 69.90 μM pNP produced) phosphatase activity. However, cluster II contained bacterial strains having high acidic phosphatase activity (40.0 to 91.82 μM pNP produced) and no siderophore production. Bacterial strains of cluster I and II showed almost similar amount of auxin production. Cluster III comprised of high auxin producing (30.87 to 59.92 μg/mL) and biofilm forming bacteria (3.68 to 12.00 O.D.) with very low phosphate solubilizing and acidic phosphatase activity. Bacterial strains of cluster III and IV showed similarity in auxin production; however, they varied in phosphate solubilization and siderophore production traits. Bacterial strains of cluster IV showed highest P-solubilization (12.64 to 42.86 μg/mL) and siderophore production ability (0.3 to 1.1 cm zone). Among all the groups formed, cluster IV contained the maximum number of bacterial strains thereby depicting the strong correlation between P accumulating trait of bacteria with P solubilization, siderophore, and auxin production.

### Abiotic stress tolerance in phosphate accumulating bacterial strains.

To assess the abiotic stress tolerance of selected PAB, all bacterial strains were grown under different abiotic stress conditions of temperature, drought, and salinity. Qualitative assessment showed that all the bacterial strains survived under imposed salinity and temperature stress up to 10 days of incubation (Table S5 and S6; https://github.com/ssrivastava-nbri/Supplementary-file/blob/main/Supplementary%20file.pdf). However, only six bacteria (SSNBRI 5, SSNBRI 11, SSNBRI 13, SSNBRI 23, SSNBRI 33, and NBRI RAR) withstand the 45% PEG simulated drought stress condition (Table S7; https://github.com/ssrivastava-nbri/Supplementary-file/blob/main/Supplementary%20file.pdf).

### Diversity based on 16S rRNA and *rpoB* genes.

**FIG 2:**
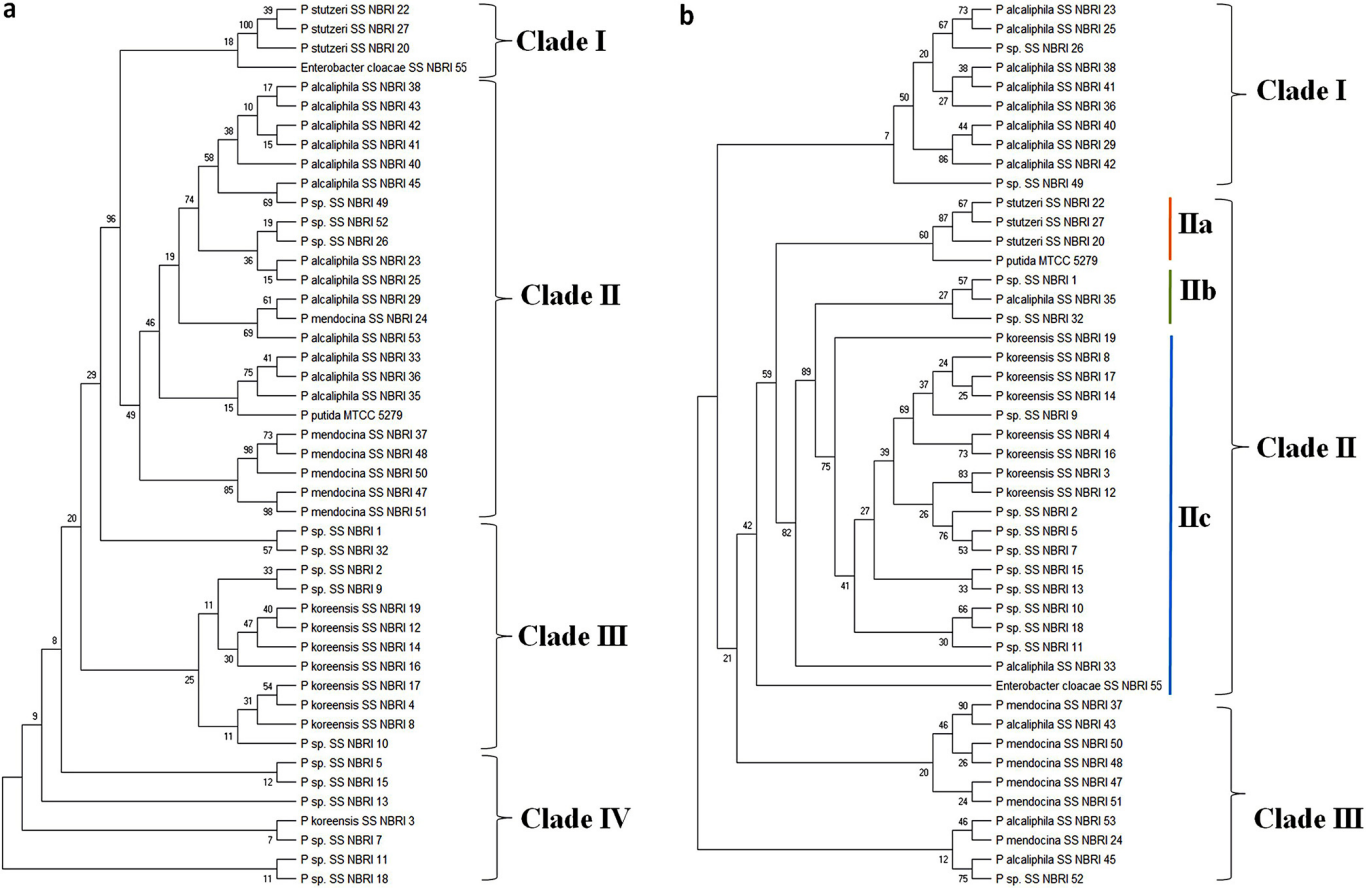
Phylogenetic tree constructed using maximum parsimony method with partial sequences of 16S rRNA (a) and *rpoB* (b) gene. The tree was created using MEGA X software. The bootstrap consensus tree inferred from 500 replicates is taken to represent the evolutionary history of the taxa analyzed.

16S rRNA gene amplification revealed that bacterial strains selected as high PAB notably belonged to genus Pseudomonas except one bacterium which was identified as Enterobacter cloacae Fig. 2a; Table S3; https://github.com/ssrivastava-nbri/Supplementary-file/blob/main/Supplementary%20file.pdf). The sequences were classified to species level having >98% similarity level. Among Pseudomonas spp., most of them were identified as *P. alcaliphila* (13), P. mendocina (7), P. stutzeri (3), *P. koreensis* (8), Enterobacter cloacae (1), and some strains (16) of Pseudomonas remained unidentified up to species level.

Genetic diversity of PAB was studied using 16S rRNA and *rpoB* gene-based phylogeny (Fig. 2a and 2b). 16S rRNA gene-based individual trees showed grouping of bacterial strains in four different clades. Clade I comprised of *P. stuzeri* (SSNBRI 22, 27 and 20) along with E. cloacae as an outgroup. While in the *rpoB* sequence based phylogenetic tree, clade IIa was comprised of P. stutzeri, outgrouped by reference strain P. putida (MTCC5279) (Fig. 2b). Bacterial strains identified as *P. alcalphila* and P. mendocina were grouped in one large clade (clade II), which was divided in two different subclades in a 16S rRNA gene based tree (Fig. 2a). These bacterial strains clustered in two different clades *viz.* clade I (*P. alcaliphila*) and III (P. mendocina) in *rpoB* gene-based phylogenetic tree (Fig. 2b). *P. koreensis* along with other unidentified strains grouped together in both 16S and *rpoB* gene-based phylogenetic tree.

### Polyphosphate kinase and exopolyphosphatase gene-based diversity.

Polyphosphate kinase (*ppk*) and exopolyphosphatase (*ppx*) are principal enzymes responsible for synthesis and degradation of polyphosphate chain. Both functional genes (*ppk* and *ppx*) were amplified in all selected PAB isolates. A single band of ∼400 bp was observed after PCR amplification of the *ppk* region and ∼480 bp of *ppx* region of each isolate.

**FIG 3:**
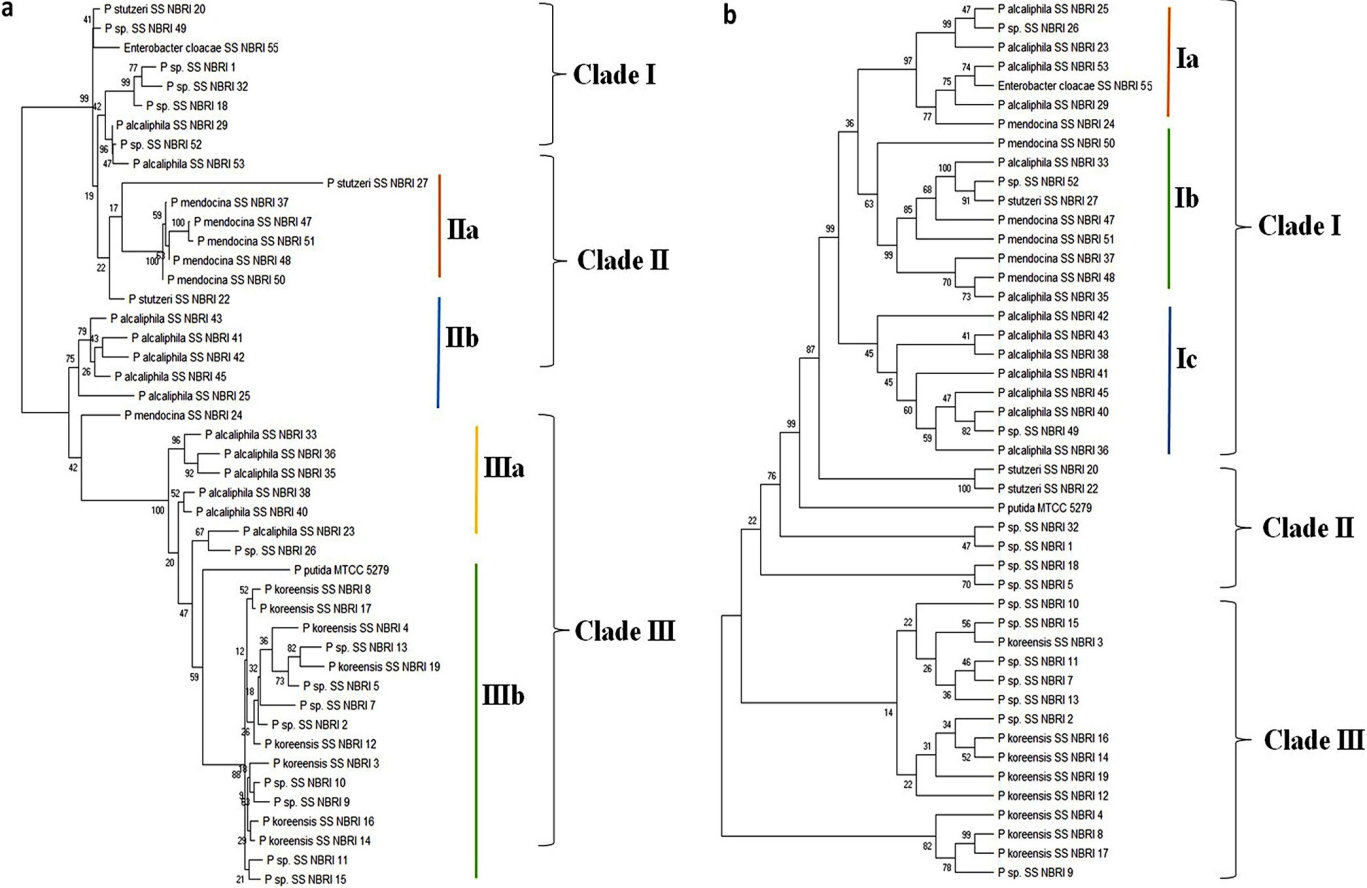
Phylogenetic tree constructed using maximum parsimony method with partial sequences of exopolyphosphatase (*ppx*) (a) and polyphosphate kinase (*ppk*) (b) gene. The tree was created using MEGA X software. The bootstrap consensus tree inferred from 500 replicates is taken to represent the evolutionary history of the taxa analyzed.

The phylogenetic tree generated using sequences of *ppk* gene of selected isolates was segregated into three clusters comprising of nine (cluster I), 12 (cluster II), and 24 (cluster III) sequences of different bacterial isolates (Fig. 3a). Cluster III, the biggest cluster, was further divided into two subgroups. Subgroup IIIa comprised of six isolates of *P. alcaliphila* isolated from different sites, while subgroup IIIb was composed mostly of *P. koreensis.* Reference strain P. putida forms the outgroup of the subgroup IIIb. Subgroup IIa and b mainly comprised of P. stutzeri and *P. alcaliphila*, respectively. Another subgroup, subgroup I, was comprised of nine heterogeneous groups of P accumulators *viz.* P. stutzeri, *P. alcaliphila*, and other Pseudomonas species (Fig. 3a).

Depending on phylogenetic distance, *ppx* genes were categorized in three major clusters (Fig. 3b). Two major clusters, i.e., cluster I and III comprised of 24 and 15 Pseudomonas spp., respectively. Species-specific clustering of *P. koreensis* along with other Pseudomonas isolates have been found in cluster III from NBRI 2 to 19 belonging to the Deokhera site. Cluster II, the smallest cluster, was comprised of reference strain P. putida along with P. stutzeri and Pseudomonas spp. (NBRI 1, 5, 18 and 32) irrespective of their isolation site. Cluster I has been found as the most diverse group comprised of P. mendocina, *P. alcaliphila*, P. stutzeri along with unidentified Pseudomonas spp. with an outgroup of *P. koreensis* at boot strap value of 89.

### Composite phylogeny tree analysis of housekeeping and functional genes.

**FIG 4:**
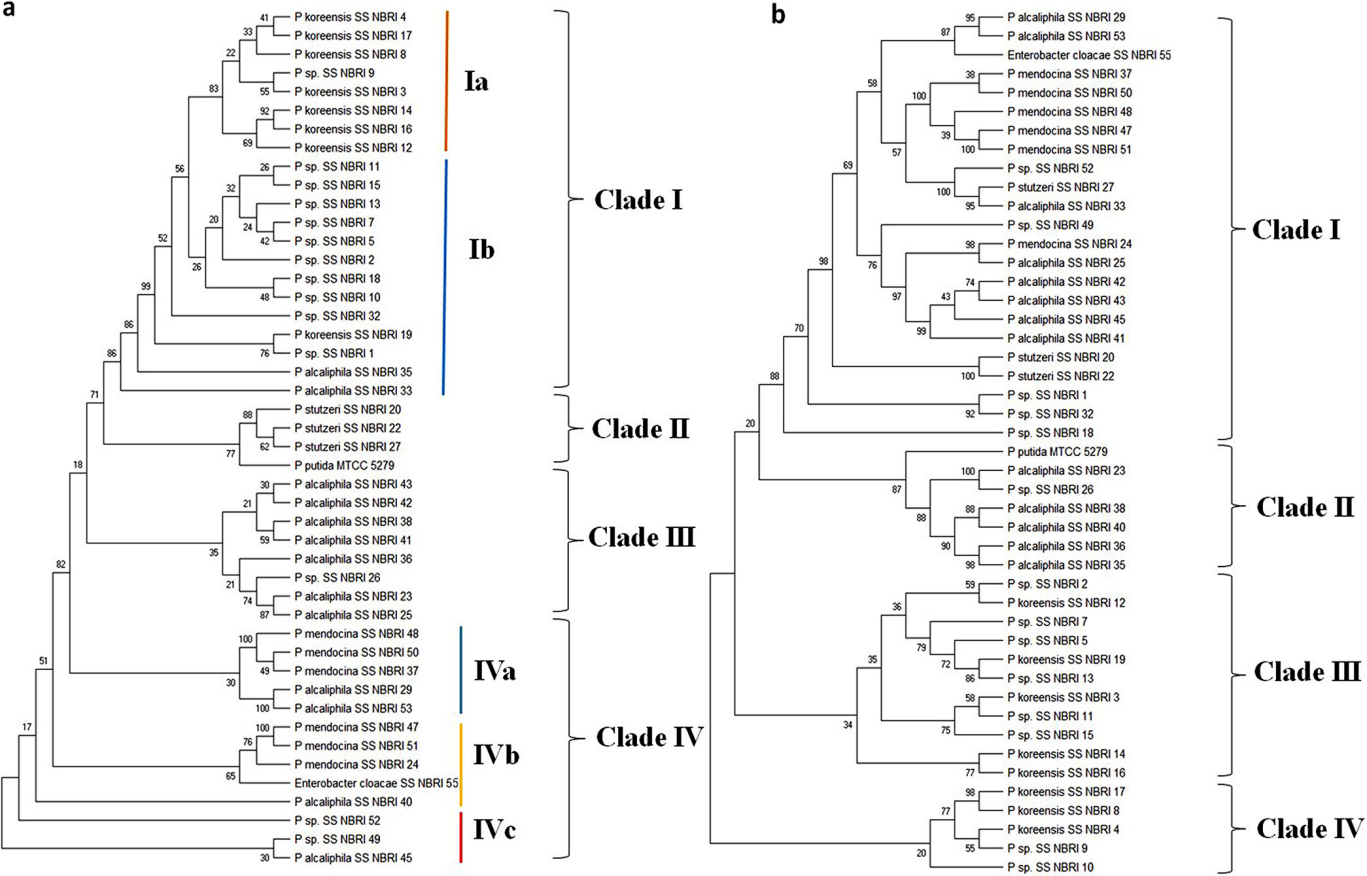
Concatenated phylogenetic tree constructed using maximum parsimony method with partial sequences of 16S rRNA and *rpoB* (a) and *ppk*-*ppx* (b). The tree was created using MEGA X software. The bootstrap consensus tree inferred from 500 replicates is taken to represent the evolutionary history of the taxa analyzed.

Aligned sequences of 16S rRNA, *rpoB*, and functional gene for polyP accumulation, i.e., *ppx* and *ppk*, were used to construct concatenated phylogeny tree of Pseudomonas spp. for a statistically comparable accuracy under a range of sampling and tree depth conditions (Fig. 4a and b). Phylogenetic analysis of aligned sequence of 16S rRNA and *rpoB* revealed variation in relationship among different Pseudomonas spp. between individual and concatenated trees (Fig. 4a). Concatenated 16S-*rpoB* tree was divided into four major clusters. All *P. koreensis* were found to be clustered in clade I along with some species of Pseudomonas. Pseudomonas spp. associated with Clade I was isolated from Deokhera except SSNBRI19 and 32 (isolated from Gujarat). P. stutzeri of different isolation sites along with reference strain P. putida formed the cluster II in the combined marker analysis. Both individual and concatenated tree showed close association of P. putida strain with P. stutzeri. Cluster III and IV mostly constituted *P. alcaliphila* and P. mendocina, respectively. Concatenated tree of 16S rRNA and *rpoB* showed species-based distribution of bacterial strains in different clades.

Another phylogenetic tree constructed based on the *ppk* and *ppx* concatenated sequences using *ppk* and *ppx* gene revealed clustering of the 46 P-accumulating strains into five major clusters (Fig. 4b). Cluster I was found to be a highly diverse group comprised of P. mendocina, *P. alcaliphila* from Punjab and Bulandshahr, and P. stutzeri from Gujarat and Punjab origin. Cluster II is a homogenous cluster of *P. alcaliphila* majorly from Bulandshahr except SSNBRI23 and 26 from Punjab. Reference strain P. putida forms outgroup of cluster II at bootstrap value 87. Cluster III included *P. koreensis* isolated from the same site (Deokhera) and unidentified Pseudomonas isolates. Cluster IV is a small cluster of five strains comprised of three *P. koreensis* strains along with SSNBRI 9 and 10 belonging to same isolation site (Deokhera). Similar interpretation can be made for cluster I and II, probably because they showed both location and species specific clustering.

### Stress tolerance and plant growth promotary attributes.

#### (i) Plant growth promotary effect of PAB on host plant *A. thaliana*.

Efficacy of PAB grouped in different clusters (Fig. 1) was evaluated for their potential to improve P status of the plants (Fig. S3 and 4; https://github.com/ssrivastava-nbri/Supplementary-file/blob/main/Supplementary%20file.pdf). Results showed that bacterial strains belonging to cluster I, III, and IV (SSNBRI 5, 11, 13, 23, 33, NBRI RAR) significantly improved P content level compared with the control. However, plants inoculated with SSNBRI 41 and SSNBRI 43 strains (cluster II) showed almost similar P content compared with control (Fig. S4; https://github.com/ssrivastava-nbri/Supplementary-file/blob/main/Supplementary%20file.pdf).

#### (ii) Stress tolerance of selected microbes.

Phosphate-accumulating bacteria tolerant to different abiotic stress (salt, temperature, and drought) along with PGP traits and ability to improve P uptake in plants (SSNBRI 5, 11, 13, 23, 33, NBRI RAR) were selected for growth kinetic study under stressed conditions of temperature (40°C), PEG simulated drought (45% PEG), and salinity (0.5 M NaCl) (Fig. S5; https://github.com/ssrivastava-nbri/Supplementary-file/blob/main/Supplementary%20file.pdf). Results showed that all the bacterial strains survived under salinity stress up to 10 days. All selected bacterial strains showed exponential growth under 0.5 M NaCl condition, except SSNBRI 33, which showed extended lag phase till the 7^th^ day. However, under temperature (40°C), stressed condition SSNBRI 23, 33, and NBRI RAR survived up to 10 days, while the other three strains attained death phase on the 3^rd^ and 5^th^ day of incubation. Similarly, under 45% PEG condition temperature tolerant SSNBRI 23 and NBRI RAR did not withstand the PEG simulated drought stress condition and attained death phase after the 2^nd^ and 5^th^ day of incubation. However, SSNBRI 13, 33, and 11 survived up to the 10^th^ day under 45% PEG simulated drought stress conditions.

#### (iii) Effect of Salinity stress on plant growth promotary traits.

Growth kinetics study of PAB under different stress conditions showed their highest tolerance to salinity stress compared with the other two tested stresses (drought and temperature). Therefore, plant growth promotary traits of selected bacterial strains were determined under NaCl-induced salinity stress condition (Fig. S6; https://github.com/ssrivastava-nbri/Supplementary-file/blob/main/Supplementary%20file.pdf). Among all the tested bacteria, SSNBRI 13, SSNBRI 33, and NBRI RAR showed higher auxin production under both control and salinity conditions compared with other three strains. At 24 h, a major decline in auxin level was noticed; however, at later days of incubation, auxin production was improved in all tested strains. Effect of salinity stress on P-solubilization attribute varied among different bacterial strains. P-solubilization activity of SSNBRI 5, 13, and 23 was severely affected under saline stress compared with the control condition. In contrast, SSNBRI 33 exhibited higher P-solubilizing potential after 24 h of incubation under stress condition compared with the control. Meanwhile, NBRI RAR was found to solubilize more P under saline stress condition compared with other bacterial strains. Similarly, some of the bacterial strains displayed enhanced alkaline phosphatase activity under stress compared with the control condition. Alkaline phosphatase activity of SSNBRI 5, 33, and RAR was increased after 48 h of incubation in saline medium. While, in NBRI 23 and 11, higher enzyme activity was found at 120 h. On the contrary, all the bacterial strains exhibited reduced acidic phosphatase activity under salinity stress, except NBRI RAR, with higher activity at 72 h and 120 h.

### Amelioration of salinity stress using *A. thaliana* as host plant.

**FIG 5:**
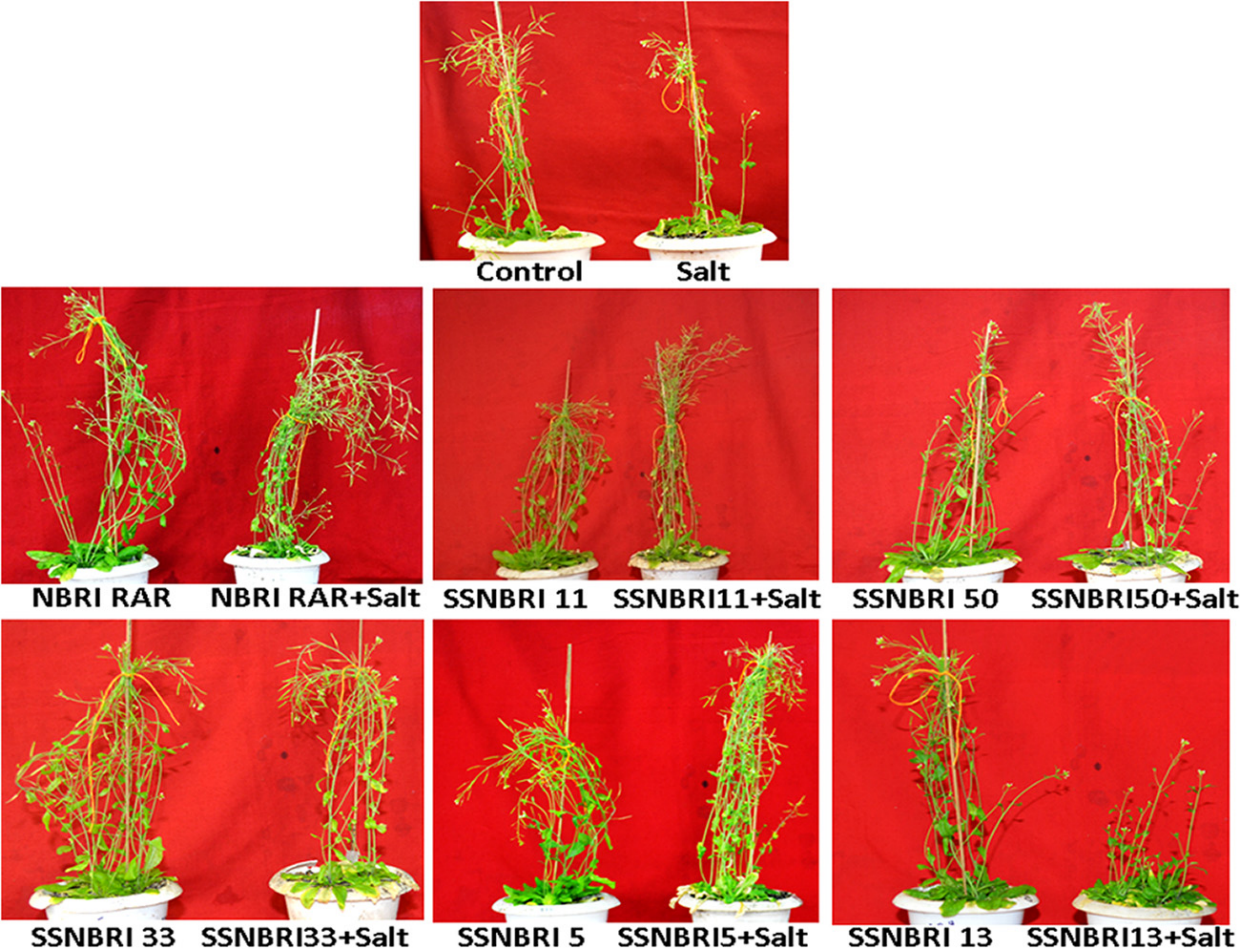
Stress ameliorating potential of phosphate accumulating bacteria on *A. thaliana* under saline condition.

Efficacy of PAB-possessing ability to improve P uptake in plants was assessed for salinity stress alleviation in *A. thaliana* as per the earlier finding of Miura et al. (28), that high phosphate accumulating mutants of *A. thaliana* has increased tolerance to salinity stress. *A. thaliana* subjected to salinity stress showed 39.13% reduction in dry weight; however, inoculation of PAB strains showed improved plant growth compared with the control (Fig. 5; Table 2). Significantly enhanced shoot and root length and dry weight were found in PAB-inoculated plants under both control and salinity stress conditions. Inoculation of bacterial strains SSNBRI 5, SSNBRI 33, and NBRI RAR showed increases in dry weight by 46.15%, 30%, and 36.36%, respectively, compared with stressed plants. Similarly, reduced number of siliques in plants grown under stress was significantly enhanced in the presence of inoculated PAB.

**TABLE 2:** Effect of P-accumulating bacteria inoculation on plant growth under salinity stressed condition^*a*^

Growth parameters	Treatment	Control	SSNBRI 5	SSNBRI 11	SSNBRI 13	SSNBRI 23	SSNBRI 33	NBRI rAR
Shoot length (cm)	Control	24.33 ± 1.42^a^	29.00 ± 2.00^c^	30.70 ± 2.22^d^	27.83 ± 1.83^b^	29.00 ± 3.00^c^	31.22 ± 1.46^d^	29.11 ± 2.01^c^
	Salinity	20.01 ± 2.17^a^	30.31 ± 1.68^d^	28.91 ± 2.79^c^	28.16 ± 2.00^c^	20.58 ± 1.92^a^	26.00 ± 1.20^b^	28.00 ± 1.30^c^
Root length (cm)	Control	5.41 ± 0.67^a^	6.65 ± 2.35^b^	5.30 ± 1.12^a^	5.18 ± 1.30^a^	8.20 ± 2.00^c^	6.68 ± 0.29^b^	6.17 ± 0.48^b^
	Salinity	5.1 ± 0.64^b^	5.00 ± 0.68^b^	3.43 ± 0.23^a^	3.83 ± 0.56^a^	5.30 ± 0.39^b^	5.51 ± 0.23^c^	6.00 ± 0.52^c^
Dry wt (g)	Control	0.23 ± 0.04^a^	0.19 ± 0.02^a^	0.28 ± 0.02^b^	0.23 ± 0.04^a^	0.22 ± 0.09^a^	0.27 ± 0.02^b^	0.26 ± 0.03^b^
	Salinity	0.14 ± 0.02^a^^b^	0.26 ± 0.10^c^	0.17 ± 0.01^b^	0.18 ± 0.02^b^	0.094 ± 0.01^a^	0.20 ± 0.04^b^	0.22 ± 0.01^b^
No. of Siliques	Control	60.20 ± 6.59^a^	71.00 ± 5.00^b^^c^	81.25 ± 17.18^d^	65 ± 10.66^b^	75.50 ± 9.50^c^	53.80 ± 10.53^a^	70.57 ± 10.31^b^^c^
	Salinity	47.8 ± 11.71^a^	90.83 ± 11.40^b^	62.33 ± 13.38^a^^b^	52.4 ± 12.12^a^	57.2 ± 10.43^a^	50.83 ± 5.36^a^	59.2 ± 14.26^a^^b^
Phosphate content (μg/mL)	Control	273.78 ± 1.3^a^	321.36 ± 2.6^a^	464.88 ± 7.28^c^	427.96 ± 16.9^b^^c^	399.88 ± 2.60^b^	398.58 ± 2.86^b^	416.78 ± 7.54^b^^c^
	Salinity	232.44 ± 8.84^a^	335.92 ± 5.2^b^	415.22 ± 4.42^c^	312.78 ± 18.34^b^	332.28 ± 1.56^b^	368.16 ± 13.92^c^	416.52 ± 4.36^c^
Sugar content (μg/mL)	Control	195.60 ± 10.80^d^	94.00 ± 12.40^a^	102.40 ± 1.20^a^	162.40 ± 16.80^c^	126.00 ± 9.20^b^	124.80 ± 1.60^b^	108.80 ± 1.60^a^^b^
	Salinity	205.60 ± 20.80^d^	133.20 ± 10.80^b^^c^	146.00 ± 1.20^c^	125.60 ± 0.42^b^^c^	111.20 ± 8.00^a^^b^	128.80 ± 1.60^b^^c^	100.00 ± 0.80^a^

a Means denoted by different letters display significant differences in treatments at *P* < 0.05.

Declined phosphate content as a negative impact of salinity stress in plants was evident compared with control. Inoculation of PAB alleviated the stress by improving the nutrient status of the plants and significantly enhanced the level of phosphate. Among different bacterial strains, SSNBRI 11 (415.22 ± 4.42 μg/mL) and NBRI RAR (416.52 ± 4.36 μg/mL) showed maximum phosphate content compared with the control. Sugar content was found to be declined in PAB-inoculated plants irrespective of treatment compared with the control plants.

## DISCUSSION

Microorganisms, the integral component of soil, are global change drivers with significant potential consequences on ecosystem functions. They are known to have different plant growth promoting traits such as phosphate solubilization/mineralization, production of plant growth hormones, and biofilm formation. The role of microorganisms in solubilization of unavailable form of phosphate in soil is well known (30). Diversity of gene *gcd* (glucose dehydrogenase); major determinant of bioavailable soil P involved in P solubilization has been previously reported (30). Additionally, P uptake, its accumulation as a reserve material and utilization under starved conditions, is a known mechanism in microbes (7). However, abundance of polyphosphate accumulating microbes and their functional and genetic diversity in soil is still obscure. Therefore, to explore this facet, the present study involves the screening and characterization of PAB from rhizospheric soil and evaluation of their genetic and functional phylogenetic relationships. Isolation of PAB from wastewater, human intestine, and oral cavity has already been established (31, 32). However, the present study is the first to report PAB from rhizosphere soil. A pretty good idea about their phylogenetic relatedness through amplicon sequencing of the 16S rRNA and functional genes *ppk* and *ppx* has been found.

### Occurrence and distribution of phosphate accumulating bacteria in soil.

Presence of relatively higher PAB in soil of varied extent, monitored through a developed screening system, emphasizes their critical role in soil P cycling (33, 34). Abundance of these PAB in soil is probably associated with the maintenance of mineral, as reported for their role in P concentration maintenance of oral cavity (9). Study showed concurrence between soil phosphorus level and abundance of high phosphate accumulating bacteria, as evident through the presence of a maximal number of high PAB in soil of Deokhera (site 2; 13 isolates) and Bulandshahr (site 3; 8 isolates) having comparatively higher P to other soil samples (Table 1; Table S1; https://github.com/ssrivastava-nbri/Supplementary-file/blob/main/Supplementary%20file.pdf). These results depict the contribution of phosphate accumulating microbes in enhancing the P pool of soil. Previous study has also reported the potential role of soil microbes in improving the bioavailability and mobilization of key nutrients in soil (34). In contrast, higher available P level of Raebareli was not sufficient to accommodate high PAB, presumably owing to high EC content of soil, as evident from earlier studies that EC has a strong correlation with P, and higher EC contributes to high P availability (35). Nonetheless, the present study did not find any strong correlation between available N, K, S, and MBC level of soil with PAB abundance. Previous studies have reported that organic carbon, pH, and P levels of soil regulate the abundance of culturable P solubilizing microbes (30, 36). The present study reports the evidence of culturable PAB in soil for the first time. However, results have not been conclusive enough in determining the factors controlling their abundance in soil. These results suggest several avenues for future research to investigate different factors affecting the abundance of PAB in soil.

### Linkage between polyP accumulation with plant growth promotion.

The contribution of rhizospheric microorganisms in mobilization and immobilization of P has been known from decades (37). Microbes with P solubilization and mineralization attributes are characterized for different plant growth promoting traits (38). The present study showed a strong correlation of P accumulating potential of bacterial strains with P solubilization, siderophore, and auxin production. However, PAB also exhibited other plant growth promoting traits such as biofilm formation, alkaline, and acidic phosphatase activity (Fig. 1, Table S4; https://github.com/ssrivastava-nbri/Supplementary-file/blob/main/Supplementary%20file.pdf). Phosphatase enzymes catalyze the hydrolysis of both phosphomono and diesters, which play important roles in plant growth under conditions of unavailable P. In bacteria, phosphatases are the part of the *pho* regulon which imports number of genes *viz.* acidic and alkaline phosphatases, high affinity P transporters, *ppk*, and *ppx*. Earlier studies have shown the association of *ppx* gene with plant growth promotary traits such as biofilm formation and motility in Campylobacter jejuni (39). Interaction of PAB grouped in different clusters (PCA plot) with *A. thaliana* showed differential response in mediating P availability to the plants. Bacterial strains belonging to clusters I, III, and IV in PCA plot (Fig. 1) improved the P status of the *A. thaliana* plants. However, no significant changes in P content of plants inoculated with bacterial strains of cluster II may be attributed to their low P solubilization and alkaline phosphatase activity (Fig. S4; Table S4; https://github.com/ssrivastava-nbri/Supplementary-file/blob/main/Supplementary%20file.pdf).

### Diversity and phylogeny of the housekeeping genes.

Phylogenetic analysis of the selected microbes using 16S rRNA and *rpoB* gene sequences revealed Pseudomonas as the predominant PAB in soil and confirms the high level promiscuity of this genus. Tobin et al. (40) has reported polyP accumulation potential in different species of Pseudomonas. In addition, other genera such as Acinetobacter spp., Microlunatus phosphovorus, *Lampropedia* spp., and *Rhodocyclus* have been reported as P accumulator in wastewater treatment plants (13).

Because 16S rRNA gene marker-based phylogenetic comparisons showed the similarity between different orthologues close to 98% to 100%, hence in order to have more sensitive and discriminating parameters, *rpoB* (RNA polymerase β-subunit), a single copy gene has been chosen as a more suitable trait to ascertain the significance of small differences (41). The present study did not show any changes at species level, when both 16S rRNA and *rpoB* sequences were compared; however, the difference was observed in their phylogenetic distribution. Nodes of 16S rRNA trees were supported by lower bootstrap values, while, *rpoB* sequence-based phylogenetic trees showed multiple nodes with higher bootstrap values, as reported by Devulder (42). 16S rRNA gene copies of some bacterial strains such as *P. alcaliphila* and P. mendocina do not form monophyletic clades compared with *rpoB* tree. Case et al. (43) reported similar distribution pattern of *Firmicutes* in 16S rRNA and *rpoB* gene-based trees.

Multilocus sequence analysis-based concatenation of housekeeping gene (16S rRNA-*rpoB)* sequences showed three clusters, with the largest heterogenous cluster II of three subgroups. Consistent with previous findings, the branches in concatenated trees are not supported by their underlying individual gene trees probably due to lateral gene transfer, very common in prokaryotes (44).

### Diversity and phylogeny of the genes encoding polyphosphate metabolism.

Polyphosphate metabolism in microbes involves some common genes such as *ppk*, *ppx*, polyphosphate glucokinase, and phosphonotransferases (45, 46). Among them, a major role is played by *ppk* and *ppx* in bacteria. Functional gene characterization study based on amplicon sequencing of *ppk* and *ppx* reveals the ubiquitous distribution of these functional genes in natural environments. These results illustrate the importance of *ppk* and *ppx* gene in bacterial growth and metabolism. Distribution of *ppx* gene in phylogenetic tree reveals its conservation at species level. Formation of distinct clusters due to uniqueness of *ppk* indicate the environmental factors mediated modification of the *ppk* genes across a variety of Pseudomonas species (Fig. 4). Similar results have also been reported earlier that the distribution of *pho*D gene of phosphate regulon is affected by environmental factors (47).

### Inconsistent distribution of *ppk* and *ppx* with the housekeeping genes.

Dealing with the incongruency of the individual gene trees of the selected P accumulating microbes, concatenated data-based phylogenetic tree emphasized the role of lateral gene transfer (LGT) as a natural mechanism of genetic diversity. Thus, the taxonomic position of P accumulating Pseudomonas is still not well defined. In this study, the *ppx* and *ppk* gene does not show consistency with the core (housekeeping) gene phylogenies (Fig. 4a and b). This suggests that *ppk* and *ppx* genes have accumulated several changes in the Pseudomonas species depending on their environmental conditions.

### Abiotic stress tolerance in phosphate accumulating bacteria and ability to ameliorate salinity stress in *A. thaliana*.

Microbes accumulate P as phosphate and energy reserve in the form of polyp granules. These granules are degraded under starved conditions and released energy is utilized for production of vital molecules, which allows them to withstand extreme environmental conditions (46). The present study showed the tolerance of all selected PAB to salinity and temperature stress. Similarly, earlier studies have reported resistance of P accumulating microbes to different environmental stresses such as nutrient limitation, salinity, heat, heavy metals, and pH (48). Among 46 PAB selected for characterization, SSNBRI 5, 11, 13, 23, 33, and NBRI RAR were tolerant to all tested abiotic stress conditions *viz.* temperature, salinity, and drought. These strains were clustered together in phylogenetic trees associated with an exopolyphosphatase gene (Fig. 3a). Presumably, exopolyphosphatase gene is involved in stress tolerance mechanism in bacteria. These results suggest that clustering of soil bacteria in phylogenetic tree are based on environmental selection of functional traits conferring stress tolerance abilities (49).

Salinity stress results in nutritional imbalances which affect the plant growth as well as yield; hence, it has been identified as constraint of global magnitude (50). P fertilization is recommended to reduce the impact of salinity on P availability; however, limited availability of P fertilizer in affected soil is one of its major drawback (51). Therefore, microbial intervention is an effective and economical way to combat salinity mediated reduced P availability in soil. The present study reports declined growth and P content of the plants under salinity stress compared with the control. Interaction of beneficial microorganisms was previously reported to alleviate salinity stress by improving vegetative growth of the plants (52). However, lower dry weight of *A. thaliana* inoculated with SSNBRI 5, 11, and 13 may be accredited to their less auxin production ability compared with other strains. Meanwhile, under saline stress condition dry weight was significantly improved in the presence of selected strain, except SSNBRI 23. This may be attributed to the maximum reduction of auxin production and P solubilization activity in SSNBRI 23 compared with the control condition (Fig. S6; https://github.com/ssrivastava-nbri/Supplementary-file/blob/main/Supplementary%20file.pdf). Microbes are well known for producing phosphatase enzymes and mediating P availability to the plants under saline stress condition (52). We also found improved P-content in plants treated with bacterial strains compared with uninoculated stressed plants. Yue et al. (53) reported improved productivity of wheat by mediating phosphate availability to the plants under stress conditions through phosphatase enzymes activity. Similarly, NBRI RAR inoculated plants showed approximately similar P level under both control and salinity conditions (Table 2), presumably due to its higher alkaline and acidic phosphatase activity in both conditions (Table S3; https://github.com/ssrivastava-nbri/Supplementary-file/blob/main/Supplementary%20file.pdf). Phosphatase enzymes have also been shown to be important for improving P mineralization in floodplain soils to sequester anthropogenic P for potentially increasing P fertility and/or reducing the P sequestration capacity of floodplain soils (54). Sugar plays an important role in stress alleviation in plants by involving different mechanisms *viz.* osmoprotection, scavenging reactive oxygen species and carbon storage (55). Higher accumulation of sugar in salinity-stressed plants was evident in the present study, as reported earlier by Parida et al. (56). Meanwhile, lowering of sugar accumulation in PAB inoculated plants emphasized their role in stress alleviation in plants. Therefore, improved yield and P status of plants in the presence of PAB indicates the efficient role of polyP accumulating microbes harboring different plant growth promotary traits in salinity stress amelioration with an ability to improve plant growth parameters.

### Conclusion.

Here we report the first study on characterization and diversity of PAB in rhizosphere soil. The promiscuity of Pseudomonas spp. as high P accumulator in soil and their diverse clustering indicates genomic differences at species level. Clustering of P. mendocina and *P. alcaliphila* in similar clades for the functional genes (*ppk* and *ppx*) predicts the presence of common ancestors in the evolutionary history. This analysis illustrates the acquisition of functional genes linked to P accumulation in different species governed by environmental conditions. Ubiquitous occurrence of *ppk* and *ppx* in all culturable PAB divulge the contribution of these microbes in soil P cycling. Additionally, association of abiotic stress tolerance with other plant growth promotary traits in PAB further extended the study by exploring the efficacy of PAB in alleviation of abiotic stress in plants. Present study of the circumvent of routine culture-dependent method led to the advent of the novel role of P accumulating microbe for plant growth promotion and stress mitigation in plants. Henceforth, efficacy of these microbes for improving phosphate availability to the plants is well justified. This study will be beneficial in exploring the detailed mechanism of PAB-mediated plant growth promotion and stress alleviation.

## MATERIAL AND METHODS

### Sampling sites and soil characterization.

Rhizosphere soil samples of different crops from cultivated fields were collected from different parts of India considered as high P input sites (Table S1, https://github.com/ssrivastava-nbri/Supplementary-file/blob/main/Supplementary%20file.pdf). Electrical conductivity and pH of soil samples was estimated in milli Q mixed with soil (1:5 ratio, soil:water) using digital conductivity (EdgeEC, Hanna, Romania) and pH meter (7310P, WTW, Germany). Available nitrogen was quantified by estimation of liberated ammonia with alkaline potassium permanganate (57). Available phosphorus and potassium in soil was estimated as reported earlier (58, 59). MBC in soil was estimated as per the protocol of Vance (60).

### Qualitative and quantitative screening of phosphate accumulating bacteria.

Heterotrophic bacterial populations were isolated from rhizosphere soil by serial dilution plating method. A detail of total bacterial and fungal population is given in Table S2 (https://github.com/ssrivastava-nbri/Supplementary-file/blob/main/Supplementary%20file.pdf). Randomly picked individual colonies were maintained on nutrient agar plate and qualitative screening of PAB was performed by inoculating the bacterial isolates in NBRI-PA-TBO media as per our lab protocol (33). Inoculated tubes were incubated at 28°C for 10 days and results were recorded at different time intervals compared with an uninoculated media control. Bacterial strains able to grow in NBRI-PA media with an ability to decolorize the toluidine blue-O dye in a period of 48 h were selected as P-accumulator.

Selected bacterial strains were inoculated (@1%, ∼10^5-6^ CFU/mL) in 150 mL Erlenmeyer flasks containing 50 mL NBRI-PA media (devoid of TBO dye) and incubated at 28°C for 48 h in a temperature-controlled incubator shaker (28). Bacterial biomass was harvested by centrifugation at 10,000 rpm for 10 min at 4°C. Extraction of Pi from bacterial cells was performed as per the protocol of Rao et al. (61). In brief, harvested bacterial cells were resuspended in 1 mL of solution I (0.145 M NaCl containing 1 mM NaF; pH 9.8). The mixture was vortexed vigorously and centrifuged at 10,000 rpm for 5 min. Supernatant was discarded and residual pellet was mixed with sodium hypochlorite (1 mL; 4%) for cell lysis. The mixture was incubated for 1 h at RT, and after, incubation supernatant was removed by centrifugation. The bacterial pellet was washed with solution II (1.5 M NaCl, 5 mM EDTA and 1 mM NaF [pH 4.6]) and finally resuspended in solution III (0.154 M NaCl [pH 7.0]). Solution III was an extraction solution, which contained P, accumulated within microbial biomass.

Extracted Pi in the supernatant was estimated by molybdenum blue method as described earlier by Muyima and Cloete (62). Extracted P (100 μL) was mixed with equal volumes of 2 N HCl, followed by hydrlysis at 95°C for 30 min in a boiling water bath followed by addition of 700 μL of molybdenum blue solution (0.42% ammonium molybdate in 1 N sulfuric acid and 10% sodium ascorbate mixed in 6:1 ratio). Reaction mixture was diluted to 5 mL and tubes were incubated at 45°C for 20 min. Final absorbance was taken at 820 nm using UV-Vis spectrophotometer. Standard was prepared using KH_2_PO_4_ and accumulated Pi was calculated in terms of biomass.

### Characterization of bacterial strains for abiotic stress tolerance and plant growth promotary attributes.

Selected bacterial strains (on the basis of high P accumulating attribute) were characterized for different plant growth promoting traits. Phosphate solubilization was estimated using NBRI-P media according to the protocol of Nautiyal (63) and Fiske and Subbarow (64). Indoleacetic acid (IAA) production and biofilm formation was performed as per the protocol of Bric et al. (65) and Srivastava et al. (66). Siderophore production was assessed qualitatively using chromazural S (CAS) assay as per the protocol of Meyer and Abdallah (67).

Initially, abiotic stress tolerance of these strains was determined qualitatively by growing bacterial cultures in nutrient broth (NB) medium under different stress conditions *viz.* temperature (45°C), drought (45% PEG), and salt (0.5 M). Based on abiotic stress tolerance and PGP traits, six bacterial strains were selected for further assessment of growth kinetics under different stress conditions. Viable cell count of bacterial strains the survived under all provided stress conditions was determined through CFU, under aforementioned stressed conditions using the serial dilution plating method. The experiment was performed up to 10 days of incubation. Additionally, PGP traits (auxin production, phosphate solubilization, alkaline, and acidic phosphatase) of these selected strains were also evaluated under salinity stress.

### PCR amplification and sequencing of 16S rRNA and *rpoB* genes.

Genomic DNA was extracted from bacterial isolates using Qiagen DNA minikit as per the manufacturer’s instruction. Identification of selected bacterial strains was performed using 16S rRNA gene sequencing as described earlier by Nautiyal et al. (68). The 16S rRNA gene was amplified using forward 27F 5′-AGAGTTTGATCCTGGCTCAG-3′ and reverse 1492R 5′-ACGGGCGGTGTGTAC-3′ primer pairs. PCR conditions involved initial denaturation for 5 min at 95°C followed by amplification for 35 cycles (30 s at 95°C, 1 min at 52°C, and 2 min. at 72°C along with a final extension of 10 min at 72°C) in Agilent sure cycler 8800. Fragment of *rpoB* gene was amplified using forward 5′-ATCTAYCGSATGATGCGYCC-3′ and reverse 5′-GTTGTTCTGGTCCATGAACTG -3′ primer pairs. PCR conditions were as follows: initial denaturation at 95°C for 3 min followed by 35 cycle of amplification (30 s at 95°C, 30 s at 55°C, and 1 min at 72°C) and final elongation at 72°C for 10 min. Reaction was setup in a 20-μL mixture containing 50 ng DNA, 1 μL of each primer (10 μM), 1 μL dNTP’s (2.5 mM), 2 μL 10X PCR Buffer, and 0.3 μL *Taq* DNA polymerase (1 unit/μL; Genei). All PCR products were purified using QIAquick PCR purification kit (Qiagen, Germany) and sequenced using same primer pairs through Sanger sequencing.

### PCR amplification and sequencing of *ppk* and *ppx* genes.

Polyphosphate kinase (*ppk*) and exopolyphosphatase (*ppx*) gene in bacterial isolates were amplified using forward and reverse primer pairs of *ppk* (forward 5′-TACGTTGAATGCTGCAGACC-3′; reverse 5′-AGCCTTCCAGCTCCTTCTTC-3′) and *ppx* (forward 5′-AGCCTGCAAATGGGCTGCG-3′ and reverse 5′-TCGACGTGATAGCGCTCC-3′). PCR conditions were similar to *rpoB* gene amplification. All PCR products were purified using QIAquick PCR purification kit (Qiagen, Germany) and sequenced using gene specific primers through Sanger sequencing. Sequences of 16S rRNA, *rpoB*, *ppk*, and *ppx* were deposited in GenBank at National Centre for Biotechnology Information (Table S3; https://github.com/ssrivastava-nbri/Supplementary-file/blob/main/Supplementary%20file.pdf).

### Phylogenetic data analysis.

NCBI GenBank databases were used to identify closely related species among test isolates using the BLASTn program. The evolutionary history was inferred using the maximum likelihood method based on the general time reversible model. The percentage of trees in which the associated taxa clustered together is shown next to the branches. Initial tree(s) for the heuristic search were obtained automatically by applying Neighbor-Join and BioNJ algorithms to a matrix of pairwise distances estimated using the maximum composite likelihood (MCL) approach, and then selecting the topology with superior log likelihood value. The tree is drawn to scale, with branch lengths measured in the number of substitutions per site. Multiple gene data sets have been used to reconstruct more robust evolutionary relationships for the same set of species through concatenation of the gene sequences of 16S *rRNA*+*rpo*B and *ppk*+*ppx* to make explicit comparisons of their genetic and functional relatedness through formation of a super-gene alignment along with the reference strain P. putida MTCC 5279-NBRIRA (69, 70).

### Plant growth promotional assay.

To study the effect of PAB inoculation on physiological modulation in host plant, *in vivo* experiment under salinity stress conditions was performed. Surface sterilized seeds of *A. thaliana* was sown in sterile soil-rite and after stratification (72 h at 4°C), pots were transferred in a temperature-controlled growth chamber (16/8 h light/dark conditions, 22°C temperature, and 70% relative humidity). Plants were irrigated weekly with nutrient solution (OS medium). Log phase grown culture of selected bacterial strains were inoculated around the plant roots at the 4 leaf stage. After 15 days of inoculation, salt stress (200 mM) was given to the plant (twice, at a difference of 1 week) (52). Root length, shoot length, and dry weight of plants were recorded after 15 days of second salt stress.

### Effect of bacterial inoculation on biochemical status of *A. thaliana*.

Phosphate acquisition in shoot tissue of *A. thaliana* was assessed by ammonium molybdate blue method as described earlier by Tsvetkova and Georgiev (71). Plant tissue (0.1 g) was homogenized in 0.5 mL of 10% perchloric acid and supernatant obtained after centrifugation (10,000 rpm for 10 min) was mixed with acetate buffer (0.1 M, pH 5.0), followed by addition of ammonium molybdate (1%, prepared in 0.5 N H_2_SO_4_) and sodium ascorbate (1%). Final absorbance was taken at 620 nm. Total sugar in the leaf tissue was estimated by the phenol-sulfuric acid method (72). In brief, shoot tissue (0.1 g) of *A. thaliana* plants grown under different treatments was macerated in 80% methanol and incubated at 70°C for 1 h. Samples were centrifuged at 10,000 rpm for 10 min and supernatant (0.5 mL) was mixed with 0.5 mL of 5% phenol and 2.5 mL of conc. H_2_SO_4_. Final absorbance was taken at 640 nm against blank using UV-visible spectrophotometer.

### Data availability.

The raw sequence data for 16S rRNA (accession no. MT629836-MT629884), *rpoB* (MT947903-MT947951 & MT982439), *ppk* (MT947952-MT948000 & MT982437) and *ppx* (MT948001-MT948049 & MT982438) have been deposited in SRA database of the NCBI.
